# Association of Commercial-to-Medicare Relative Prices With Health System Financial Performance

**DOI:** 10.1001/jamahealthforum.2022.5444

**Published:** 2023-02-10

**Authors:** Fredric Blavin, Nancy Kane, Robert Berenson, Bonnie Blanchfield, Stephen Zuckerman

**Affiliations:** 1 Health Policy Center, Urban Institute, Washington, DC; 2 Harvard T. H. Chan School of Public Health, Harvard University, Boston, Massachusetts

## Abstract

**Importance:**

Various studies have documented the rise in commercial insurance prices during the past 2 decades; however, estimates on the association of rising costs with health systems’ financial health are lacking. This study calculated 2 measures from standardized Audited Financial Statements (AFSs)—operating margins and days of unrestricted cash on hand—to explore the associations.

**Objective:**

To estimate the association between health systems’ financial condition and the ratio of commercial to Medicare relative prices.

**Design, Setting, and Participants:**

This cross-sectional analysis combined standardized 2018 AFSs from a large sample of US health systems with publicly available relative price data to assess the association between their financial outcomes and commercial-to-Medicare relative inpatient prices. The 2018 AFSs were collected and standardized from a convenience sample of multihospital health systems and single hospitals that were included in round 4 of the RAND Hospital Price Transparency Study. Cross-sectional, multivariate regression models were estimated, controlling for payer mix and other system characteristics, and models were weighted by health systems’ 2018 adjusted admissions. The analyses were conducted July 2021 through November 2022.

**Exposures:**

The commercial-to-Medicare relative price for inpatient services (2018-2020 pooled average), which represents the average amount paid by commercial plans as a percentage of what Medicare would have paid to the same health system for the same services.

**Main Outcomes and Measures:**

Operating margins and days cash on hand, which capture complementary aspects of financial performance (profitability and liquidity).

**Results:**

The study sample included 156 health systems in the US, representing diverse geography, size, and ownership type. Mean (SD) days cash on hand were 180.1 (113.3) and operating margins were 3.3% (3.6%) in 2018. Overall, a 1-unit increase in the commercial-to-Medicare relative price ratio was associated with a 21.3% (95% CI, 21.3% to 21.4%*; P <* .001) increase in days cash on hand and a 2.7 (95% CI, 2.7 to 2.7; *P* < .001) percentage point increase in average operating margins. Higher Medicaid payer mix share was associated with fewer days cash on hand (−3.3%; 95% CI, −3.3% to −3.3%; *P* < .001) and lower operating margins (−0.081; 95% CI, −0.082 to −0.081; *P* < .001).

**Conclusions and Relevance:**

This cross-sectional study of health system financial data found that higher commercial-to-Medicare relative prices and a lower Medicaid payer share were associated with higher profits and more days cash on hand. These findings provide evidence against the claim that relatively higher commercial prices are primarily used to offset losses from public payers rather than to increase profits and liquidity.

## Key Points

**Question:**

What is the estimated association between health systems’ financial performance and commercial-to-Medicare relative prices?

**Findings:**

This cross-sectional study using standardized audited financial data from 156 health systems and a multivariate regression approach found that a 1-unit increase in the relative inpatient price ratio was associated with a 21.3% increase in days cash on hand and a 2.7 percentage point increase in average operating margins.

**Meaning:**

The findings of this cross-sectional study highlight that prices negotiated by health systems with commercial payers are an important factor in explaining differential health system profitability and wealth.

## Teaser

This cross-sectional study of more than 150 health systems assesses the association between their financial outcomes and commercial-to-Medicare relative inpatient prices.

## Introduction

Numerous studies have demonstrated the inexorable rise in commercial insurance prices for hospital services over the past 2 decades. From 1995 to 2000, commercial insurance rates averaged 110% of Medicare payments for inpatient care.^1^ Subsequent studies have shown that commercial-to-Medicare relative prices continued to rise. The most recent national study of commercial-to-Medicare prices performed by RAND researchers for 2018 to 2020^2^ found that commercial rates for combined inpatient and outpatient services averaged 224% of Medicare rates.

Quantitative studies documenting how relative commercial prices affect financial health are lacking, despite anecdotal reports and qualitative studies that strongly suggest these factors are important to understanding the reasons for differential health system wealth. Ly and Cutler^3^ studied factors associated with improved operating margins, finding that nonprofit status was associated with increases in operating margins, while rural status and having a larger share of patients covered by Medicare were associated with decreases in operating margins. There was no association between profitability and number of profitable services.

Some investigators contend that higher commercial prices are necessary to offset shortfalls from public payers—commonly referred to as cost shifting—whereas others assert that hospitals with greater market power negotiate higher prices from commercial payers, regardless of the payment levels from public payers.^4^ If cost shifting (eg, an unfavorable payer mix with a high Medicaid share of revenue) were the primary reason for high and rising prices, one might expect no association between commercial payment rates and financial health, whereas if market power was the dominant reason, there should be a positive association between commercial payment rates and financial health, as reflected in measures of profitability and liquidity.

Operating margins are an important but only partial representation of hospital financial well-being. For example, a recent study of hospitals in North Carolina^5^ demonstrated that in 2020, although operating profit margins narrowed, cash and investments skyrocketed, and total margins increased. Similarly, a national study of the association of COVID-19 with 2020 hospital finances found that although hospitals experienced a sizeable reduction in operating income from patient care only, excluding COVID relief grants, their overall profit margins from all sources remained similar to those of prior years.^6^

For this study, we combined price and payer mix data with 2 complementary measures from Audited Financial Statements (AFSs)—standardized operating margins and days of unrestricted days cash on hand (DCOH)—to quantitatively explore these associations. In essence, DCOH is a reliable measure of liquidity—it measures the number of days that health systems can cover their expenses with accumulated unrestricted cash and investments. In an earlier study,^7^ we found only a moderate correlation between operating margins and DCOH, suggesting that the 2 measures would provide complementary information on which to explore the associations between financial position and commercial prices and payer mix.

The AFSs facilitate assessment of not only profitability but also of liquidity, solvency, and other important components that together determine a health system’s financial position. Given that we previously demonstrated the feasibility of analyzing health system AFSs to provide a comprehensive picture of financial positions,^7^ we aim in this analysis to better understand the associations between health systems’ financial positions, negotiated commercial prices, and payer mix.

## Methods

This study was exempted from review by the Urban Institute’s Institutional Review Board because it did not involve human participants. The study design and reporting followed the Strengthening the Reporting of Observational Studies in Epidemiology (STROBE) reporting guidelines for cross-sectional studies.

### Data Collection

This analysis combined 2018 hospital- and system-level data from 3 sources: the RAND Hospital Price Transparency Study for prices,^2,8^ AFSs for financial measures and payer mix, and the American Hospital Association (AHA)^9^ for other hospital and health system characteristics. Table 1 summarizes the measures obtained from each data source.

**Table 1.** Data Sources and Measures Used to Assess Association of Prices With Selected Financial Ratios of Sampled Health Systems

Data source and measure	Description
**RAND Hospital Price Transparency Study** ^2,8^	
Commercial-to-Medicare price ratio	Defined as allowed amount from private health plan claims divided by Medicare allowed amount; measure was calculated for the same services provided by the same hospital, using Medicare’s price-setting formulas, and adjusted for geographic variation using Medicare wage index.
Part of multi-hospital system	Includes groups of 2 or more short-stay hospitals under joint ownership according to the Agency for Healthcare Research and Quality’s Compendium of US Health Systems.
**Audited Financial Statements**	
Operating margins	Defined as total operating revenue minus total operating expense divided by the total operating revenue. Operating margin indicates profitability from operating activities, including delivery of health care services, research, education, and other operating activities central to organizational mission. Measure excludes investment income.
Days cash on hand (from unrestricted sources)	Defined as (cash, cash equivalents, money market, short- and long-term investments, marketable securities designated as current or noncurrent, including board-designated and undesignated funds) divided by ([total operating expenses minus depreciation and amortization] divided by 365). Ratio excludes donor-restricted and legally obligated financial assets, eg, debt service or insurance reserves. It indicates the number of days a health system can cover its operating costs with its unrestricted cash and investments in financial assets without collecting additional revenue. High values are positively associated with a system’s ability to pay its current bills, access long-term debt, and generate investment income.
Payer mix	Net patient revenue share from Medicaid as a percentage of total net patient service revenue.
**American Hospital Association** ^9^	
Adjusted admissions	Defined as admissions plus (admissions multiplied by [outpatient revenue divided by inpatient revenue]); reflects the sum of admissions and equivalent admissions attributed to outpatient services. Measure of system size used to weight regressions.
Rural	Defined as share of adjusted admissions in system that are rural; a rural hospital is located outside a metropolitan statistical area, as designated by the US Office of Management and Budget.
Ownership	Predominant ownership within a system: government (nonfederal), nonprofit, or for-profit.
Region	Predominant region within a health system: Midwest, Northeast, South, and West.

#### RAND Data

We used round 3 of RAND’s Hospital Price Transparency Study (2016–2018 pooled data)^8^ to select a sample of multihospital systems and independent hospitals. The round 3 RAND data report prices for 3112 hospitals and 178 health systems identified by name in all states except Maryland. We updated the price data when round 4 (2018-2020 pooled data) of the RAND study^2^ was released in May 2022, which included spending on more hospitals (>4000 hospitals) and a greater number of claims. This analysis focused exclusively on the 2018 to 2020 pooled price data because the data were recent and based on a larger sample size of hospitals and claims.

The main study analysis used the ratio of commercial to Medicare relative price for inpatient services, meaning the allowed amount (average negotiated rate) paid by a commercial plan as a percentage of what Medicare would pay for the same service to the same health system. The RAND study used claims data from private health plans to measure and compare hospitals prices at a high level of detail. The data came from self-insured employers, select state-based all-payer claims databases, and health plans that chose to participate. The price data were constructed from both facility and professional claims for inpatient and outpatient services provided by Medicare-certified short-stay hospitals. For each private claim, RAND repriced the service using Medicare's grouping and pricing algorithms and reported price levels and trends for both hospitals and hospital systems identified by name. Systems included groups of 2 or more short-stay hospitals under joint ownership according to the Agency for Healthcare Research and Quality’s Compendium of US Health Systems.^2^

#### Audited Financial Statements

Policy analysts and researchers have historically relied on the Centers for Medicare & Medicaid Services’ Medicare Cost Reports that hospitals use to submit annual financial data. However, these financial data are unaudited, their accounting elements lack critical detail, and the reporting entity is at the facility level, not the system level, which was the focus of this analysis.^10,11,12^

Audited Financial Statements are the criterion standard of financial data^13^; they report the financial performance of all entities within a health system, ie, a parent company that has governance and financial and managerial control over member hospitals, affiliates, and subsidiaries. Made public within 3 to 6 months after the close of a health system’s fiscal year, AFSs are required of most health systems. For health systems that issue municipal bonds, AFSs are publicly available through EMMA (Electronic Municipal Market Access) for nonprofit systems and from the US Security and Exchange Commission’s EDGAR (Electronic Data Gathering, Analysis, and Retrieval) system for publicly owned for-profit systems. In addition, AFSs may be available from some states that require health systems to submit them to a state agency, and some government-owned systems post their AFSs on their own websites.

Operating margins and DCOH are 2 of the most relevant outcomes because they are reliable measures that capture complementary aspects of financial performance: profitability and liquidity. Operating margins measure the profitability from operating activities, including delivery of health care services, research, education, and other operating activities central to an organization’s mission), whereas DCOH measures the number of days for which a system can pay its operating expenses given the unrestricted financial assets reported on its balance sheet.

We calculated measures of hospital financial health from 2018 AFSs. We used 2018 AFS data because they aligned with the timing of the RAND price study^8^ and were not influenced by the COVID-19 pandemic. Overall, we identified, collected, and standardized AFSs on 141 of the 178 multihospital health systems in the RAND data. To represent hospitals that are not part of multihospital systems, we selected 15 single hospitals based on the ease of availability of their AFS data (ie, the AFSs were used in prior analyses or were publicly available on a state website). For simplicity, we will refer to the 156 observations in the study sample as health systems.

We also collected the Medicaid payer share (based on net patient revenue) from the AFSs or state websites to use as a control variable in the statistical model. A major concern was that some health systems have categorized Medicare Advantage (MA) as part a of commercial or managed care, while others (including those we were able to directly categorize) categorized MA as Medicare. As such, we did not control for the Medicare and commercial payer shares because we could not separate these sources of revenue.

Using a glossary of terms as well as a secondary review of each health system’s data to ensure reliability, the research team downloaded individual AFSs and entered elements from the AFSs into a spreadsheet template to standardize financial data and generate financial ratios that were comparable across health systems. Details of the standardization of each measure are summarized in Table 1 and described elsewhere.^7^

#### Other Hospital Characteristics

We used the 2018 AHA annual survey^9^ to provide additional information on the characteristics of the health systems in the study sample. The AHA annual survey is a voluntary survey that yields consistent and comprehensive data about hospital facilities. We used the survey to collect information on system size (adjusted admissions), ownership type (nonfederal government, nonprofit, and for-profit), and geography (region and rurality). The eAppendix in Supplement 1 compares the characteristics of hospitals in the study sample with all nonfederal, general medical, and surgical hospitals in the 2018 AHA annual survey.

We excluded for-profit systems in all models that focused on DCOH; for-profit systems have substantially lower levels of DCOH because they typically return excess cash to shareholders or owners as dividends or through stock buybacks when the cash cannot be productively invested in business assets.^7^

### Statistical Analysis

We assessed the distribution and skewness of the DCOH and operating measures variables using Shapiro-Wilk tests of normality. We used multivariate linear regressions to estimate the association between health system financial outcomes and the commercial-to-Medicare relative inpatient price, controlling for payer mix and other system characteristics. We weighted all multivariate regressions by system size (adjusted admissions) and conducted statistical tests using robust standard errors. We deliberately focused on the weighted models that emphasize associations among larger systems, which generally include more hospitals, because they are more indicative of the importance of relative commercial prices across the health care system as a whole. We described unweighted descriptive statistics in the main analysis; unweighted regression results are available in eTable 1 in Supplement 1.

The statistical analysis was performed in July 2021 through November 2022, using Stata, version 17 (StataCorp). We used 2-tailed hypothesis testing with a significance threshold of *P* < .05, unless otherwise indicated.

## Results

Based on a visual assessment of the outcome variables and findings from the Shapiro-Wilk test of normality, we rejected the null hypothesis that DCOH (*P* < .001) were normally distributed but could not reject the null hypothesis that operating margins (*P* = .35) were normally distributed. Therefore, we displayed both DCOH measures in a descriptive table but used the DCOH log transformed as a dependent variable in the multivariate models.

### Study Sample Descriptive Statistics

Table 2 includes the unweighted and weighted descriptive statistics for the study sample of 156 health systems, most (n = 141; 90.4%) of which were multihospital systems. The average (SD) operating margins from the unweighted sample was 2.9% (3.8%), and these health systems had an average (SD) of 201.4 DCOH (109.7; log transformed mean [SD], 5.1 [0.9]). These estimates were consistent with the 2018 to 2019 median values for rated bonds, which were 213 to 218 for DCOH and 2.1% to 2.7% for operating margins, based on ratings from FitchRatings and S&P Global.^14,15^

**Table 2.** Descriptive Statistics of the Study Sample of Health Systems^a^

Characteristic	Mean (SD)	
	Unweighted	Weighted
Sample size, No.	156	36 226 392
Financial outcomes^b^		
Days cash on hand	201.4 (109.7)	180.1 (113.3)
Days cash on hand, log transformed	5.1 (0.9)	4.7 (1.4)
Operating margins	2.9 (3.8)	3.3 (3.6)
Commercial-to-Medicare price ratio^c^		
Inpatient	2.3 (0.6)	2.4 (0.5)
Outpatient	2.8 (0.9)	2.8 (0.8)
Inpatient and outpatient	2.5 (0.7)	2.6 (0.6)
% Net patient service revenue Medicaid^b^	13.5 (8.8)	12.7 (6.3)
% Rural of adjusted admissions^d^	4.1 (16.5)	1.4 (4.5)
Part of multihospital system, No. (%)	141 (90.4)	35 956 592 (99.3)
Ownership, No. (%)^d^		
Nonprofit	138 (88.5)	29 038 843 (80.2)
For-profit	5 (3.2)	6 222 990 (17.2)
Nonfederal government	13 (8.3)	964 559 (2.7)
Region, No. (%)^d^		
Midwest	40 (25.6)	10 797 619 (29.8)
Northeast	51 (32.7)	7 219 989 (19.9)
South	32 (20.5)	12 823 108 (35.4)
West	33 (21.2)	5 385 676 (14.9)

^a^ Includes 156 observations covering independent hospitals (n = 15) and multihospital systems (n = 141) from round 4 of the RAND Hospital Price Transparency Study (2018-2020 pooled hospital price data).^2^ This final sample excluded entities with missing payer mix/other financial metrics from the Audited Financial Statement data. Multihospital systems included groups of ≥2 short-stay hospitals under joint ownership per the Agency for Healthcare Research and Quality’s Compendium of US Health Systems.

^b^ Collected and calculated from Audited Financial Statements.

^c^ Round 4 of the RAND Hospital Price Transparency Study.^2^ Price ratio defined as the actual private allowed amount divided by the Medicare allowed amount for the same services provided by the same health facility.

^d^ From the 2018 American Hospital Association annual survey database.^9^

Among health systems in the study sample, the average (SD) commercial-to-Medicare relative inpatient price ratio in 2018 to 2020 was 2.3 (0.6), with higher average relative prices for outpatient services; that is, employers and private insurers paid 230% of what Medicare would have paid for the same inpatient services at the same facilities. This estimate was comparable with the 2016 to 2018 average relative price from round 3 of the RAND study^8^ and slightly higher than the 2018 to 2020 average price from round 4.^2^ However, among the common data contributors in both rounds 3 and 4 of the RAND study, from which the study sample was drawn, the 2018 to 2020 commercial-to-Medicare relative price was nearly identical to the sample average. These health systems averaged (SD) a Medicaid payer share of 13.5% (8.8%) in 2018.^8^

The diverse mix of health systems-based on geography, size, and ownership type-in the study sample averaged 232 221 adjusted admissions in 2018, of which 4.1% (SD, 16.5%) were associated with hospitals in rural areas. Most (n = 138; 88.5%) of the health systems were nonprofits and accounted for 55.0% of total adjusted admissions among nonprofit hospitals in the 2018 AHA annual survey.^9^ Although for-profit health systems accounted for only 3.2% of the sample (n = 5), they represented a highly disproportionate share (67.5%) of for-profit systems’ adjusted admissions. In contrast, government-owned health systems accounted for 8.3% (n = 13) and represented 9.7% of total adjusted admissions among this ownership type in 2018.

The estimates weighted by adjusted admissions were similar to the unweighted estimates but were more skewed toward multihospital health systems, for-profit systems, and systems operating in nonrural areas and the US Midwest, and South regions. For example, nearly all (99.3%) of the adjusted admissions in the sample were attributable to health systems, but only 1.4% of adjusted admissions were from rural areas. In addition, for-profit systems represented only 3.2% of the unweighted sample but accounted for 17.2% of total adjusted admissions.

The Figure represents the association between the commercial-to-Medicare relative price and the 2 financial outcomes for each health system in the study sample (unweighted). There were clear positive associations between the commercial-to-Medicare relative price and DCOH (Figure, A) as well as operating margins (Figure, B). The correlation between the inpatient relative price and DCOH was 0.298, and between relative price and operating margins, 0.223.

**Figure.**
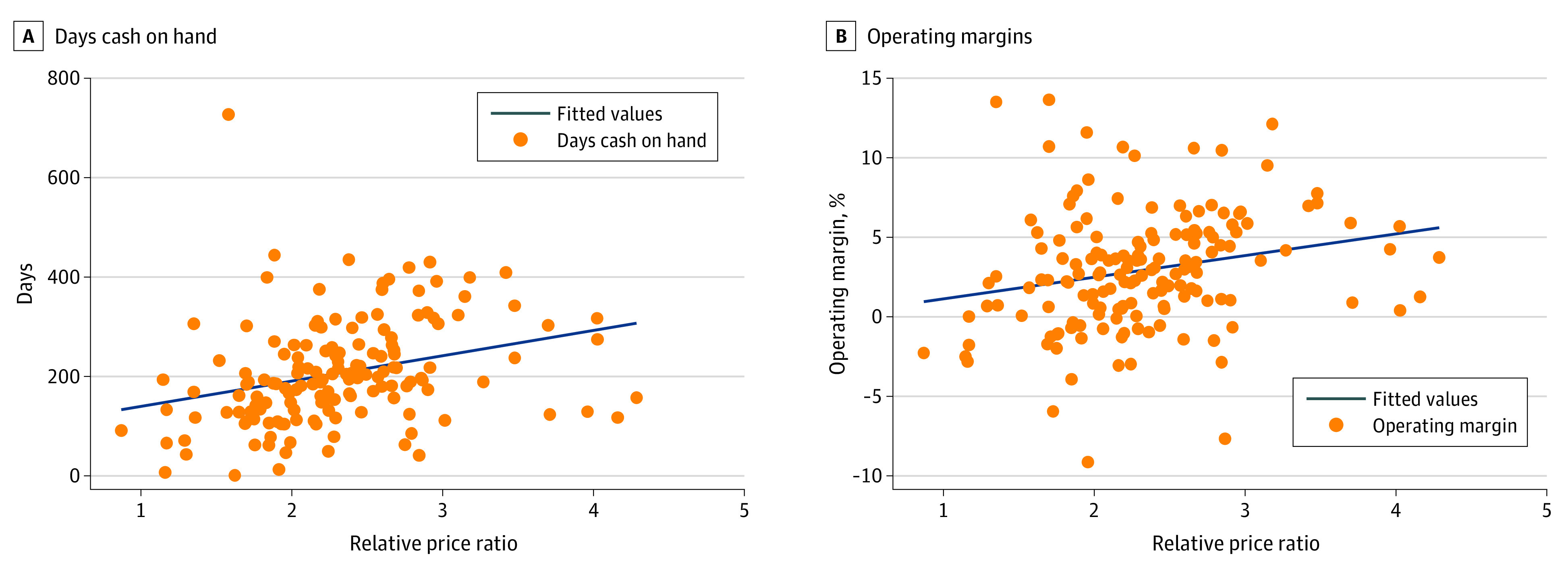
Univariate Relationship Between Commercial-to-Medicare Price Ratio for Inpatient Services and a Health System’s Financial Outcomes, Unweighted The operating margins sample includes 153 independent hospitals (n = 15) and multihospital systems (n = 138) that were included in round 4 of the RAND Hospital Price Transparency Study,^2^ the source of the price ratio data. Days cash on hand exclude for-profit systems. The price ratio is defined as the actual private allowed amount divided by the Medicare allowed amount for the same services provided by the same hospital or system for inpatient services. Financial outcomes were collected and calculated from Audited Financial Statements.

### Multivariate Findings

Table 3 includes the multivariate regression results for the DCOH model based on 151 nonprofit and nonfederal government-owned health systems, weighted by the adjusted admissions. The association between DCOH and the commercial-to-Medicare relative price was large and statistically significant, even after controlling for system status, rurality, ownership type, region, and payer mix. Overall, a 1-unit increase in the relative price ratio (eg, a price ratio increasing from 1 to 2) was associated with a 21.3% (95% CI, 21.3%-21.4%) increase in DCOH.

**Table 3.** Association of Commercial-to-Medicare Price Ratio for Inpatient Services With Health Systems’ Financial Outcomes in 2018, Multivariate Model

Characteristic	DCOH, log transformed (SD)^a^	Operating margins (SD)
Relative price for inpatient services	0.213 (0.213 to 0.214)^b^	2.71 (2.71 to 2.72)^b^
Part of multihospital system	0.294 (0.291 to 0.297)^b^	−3.63 (−3.65 to −3.61)^b^
Rural share of adjusted admissions	0.142 (0.139 to 0.145)^b^	−3.36 (−3.39 to −3.32)^b^
Ownership		
Nonprofit	[Reference category]	[Reference category]
For-profit	NA^a^	2.73 (2.73 to 2.74)^b^
Government	−0.126 (−0.127 to −0.125)^b^	1.01 (1.00 to 1.02)^b^
Region		
Midwest	−0.063 (−0.063 to −0.062)^b^	−0.85 (−0.86 to −0.85)^b^
Northeast	−0.697 (−0.698 to −0.696)^b^	−0.94 (−0.94 to −0.93)^b^
South	−0.410 (−0.410 to −0.409)^b^	−0.97 (−0.97 to −0.96)^b^
West	[Reference category]	[Reference category]
Medicaid %	−0.033 (−0.033 to −0.033)^b^	−0.08 (−0.08 to −0.08)^b^
Constant	5.158 (5.155 to 5.162)^b^	1.65 (1.63 to 1.67)^b^
Observations^c^	30 003 402	36 226 392
*R* ^2^	0.32	0.29

Abbreviation: DCOH, days cash on hand.

^a^ Estimates associated with for-profit ownership are missing because the DCOH sample excludes for-profit organizations.

^b^ *P* < .01.

^c^ Models were weighted by total adjusted admissions within the organization. Nonprofit ownership and West region were the excluded reference categories. Sample includes 156 observations covering independent hospitals (n = 15) and multihospital systems (n = 141) from round 4 of the RAND Hospital Price Transparency Study (2018-2020 pooled hospital price data).^2^ This final sample excludes entities that had missing payer mix or other financial metrics from the Audited Financial Statement data. Multihospital systems include groups of ≥2 short-stay hospitals under joint ownership per the Agency for Healthcare Research and Quality’s Compendium of US Health Systems.

We also found that higher Medicaid share of revenue was associated with fewer DCOH. A 1 percentage point (pp) increase in Medicaid payer share was associated with 3.3% (95% CI, −3.3% to −3.3%) fewer DCOH on average. Government ownership and being in the Northeast, Midwest, or South regions were associated with fewer DCOH, whereas being part of a multihospital system and higher rural adjusted admissions were associated with more DCOH.

Table 3 also shows the multivariate regression results for the operating margins model for the full sample, which includes for-profit systems (n = 156). The association between operating margins and the commercial-to-Medicare relative price was positive and statistically significant: a 1-unit increase in the relative price ratio was associated with a 2.71 (95% CI, 2.71 to 2.72) pp increase in average operating margins. Higher Medicaid payer share was associated with slightly lower operating margins; that is, a 1-pp increase in the Medicaid payer share was associated with a 0.08 (95% CI, −0.08 to −0.08) pp decline in average operating margins. For-profit and government ownership (vs nonprofit ownership) and being in the West were associated with higher average operating margins, and higher rural adjusted admissions and multisystem hospital affiliation were associated with lower average margins. The finding that the small sample of 13 government-owned systems had higher operating margins than nonprofits was not necessarily surprising because government systems often receive substantial subsidies at the state and local levels. A prior study^16^ found that government-owned hospitals in highly competitive markets were more profitable than other safety-net hospitals.

For both the DCOH and operating margins regressions, the *R*^2^ values indicate that nearly one-third of the variation in each outcome was explained by the model. When we estimated models with the combined inpatient and outpatient price measure, we also found comparable associations among each of the financial outcomes and the relative price ratio and payer mix (eTable 2 in Supplement 1). We observed comparable estimates in unweighted models, although the association between price and operating margins was smaller and estimated with less precision (eTable 3 in Supplement 1).

## Discussion

Recent quantitative studies have confirmed the anecdotal and qualitative research demonstrating the wide variation in the financial health of hospitals and health care systems, reflected in both operating performance and levels of cash and investments.^6,7,17,18^ The *haves*, a group consisting mostly of nonprofit multihospital health systems, have substantial reserves available for capital expansion and the ability to weather unexpected revenue shortfalls, such as those experienced at the onset of the COVID-19 pandemic. The *have nots*, which include some government-owned safety-net hospitals and many rural hospitals, struggle to maintain core operations and sudden revenue shocks threaten their survival.

During the COVID-19 pandemic, the financial gulf between the *haves* and the *have nots* may have only become larger. Exacerbating the underlying differences in financial position, the Provider Relief Fund’s allocation formula for the initial $50 billion distribution was based on net patient revenue in 2018, which benefited health systems that were more sufficiently resourced before the pandemic and disadvantaged safety-net systems.^19^

To our knowledge, the reasons for the large disparities in financial health across hospitals and health systems have not been clearly established, although numerous explanations have been considered. For example, some hospitals and systems may be more successful at managing costs, with leaner staffing, less generous salaries, shared overhead costs, better revenue collection, less ambitious acquisition strategies, and stronger efforts to reduce lengths of stay.^3^ However, the revenue side of the ledger is more commonly discussed as a major source of surpluses and variation in health systems’ financial health. Proffered explanations include the growing ability of some systems to attain market leverage and higher prices in negotiations with commercial insurers, given their mergers and acquisitions^20^; a more profitable mix of patients served based on patient insurance statuse^3^; and selective delivery of more profitable services.^3,21^

As a contribution to the literature, the findings from this study highlight how high commercial prices and a lower Medicaid payer share can be important factors in explaining differential health system financial health. These findings also provide evidence against the presumption that relatively higher commercial prices were primarily used to offset losses from public payers; clearly, they contributed to higher profits and greater wealth.

### Limitations

This analysis was primarily descriptive, and a health system’s financial performance may be associated with various factors that were not evaluated; eg, market concentration, risk, and demographic profiles of patients. This analysis also had sample size limitations considering the substantial time and resources needed to collect and standardize each health systems’ AFSs and to match financial data to the relative price data. Despite these limitations, the study sample captured a diverse range of systems and hospitals by ownership type, region, and size.

Although the AFS data are the criterion standard for health system financial performance, they do not provide reliably comparable payer mix data, particularly for MA, as described earlier. Some health systems do not include premium or capitation revenue as part of their net patient service revenue by payer. Given the increasing role that MA and capitation or premium revenue play in health systems, these limitations suggest the need for standardizing disclosures that specify MA and other “at risk” revenues by payer. In addition, there may be some potential measurement noise and other limitations associated with the RAND hospital price data.^2,8^ Despite these limitations, this analysis provides insight into the associations between health systems’ financial position, negotiated commercial prices, and payer mix.

## Conclusions

This cross-sectional study of health system financial data found a positive and significant association between the commercial-to-Medicare relative price and 2 complementary measures from AFSs on health systems’ financial position—DCOH and operating margins. We also found that a higher Medicaid payer mix share was associated with fewer DCOH and with slightly lower operating margins.

## Notes

Supplement 1. **eAppendix.** Comparison of Sample Characteristics to the Universe of Health Systems from the AHA **eTable 1.** Characteristics of Analysis Sample vs AHA **eTable 2.** Association Between Commercial-to-Medicare Price Ratio for Inpatient and Outpatient Services and Organization's Financial Outcomes, Multivariate Model, 2018–2020 RAND Prices **eTable 3.** Association Between Commercial-to-Medicare Price Ratio for Inpatient Services and Organization's Financial Outcomes, Multivariate Model, 2018–2020 RAND Prices (Unweighted)

Supplement 2.
Data Sharing Statement
