# Supplementary material for: Association of Commercial-to-Medicare Relative Prices With Health System Financial Performance
Source: JAMA Health Forum. 2023 Feb 10;4(2):e225444. doi: 10.1001/jamahealthforum.2022.5444 (PMC9918880; doi:10.1001/jamahealthforum.2022.5444)
Supplement: Supplement 1. — eAppendix. Comparison of Sample Characteristics to the Universe of Health Systems from the AHA eTable 1. Characteristics of Analysis Sample vs AHA eTable 2. Association Between Commercial-to-Medicare Price Ratio for Inpatient and Outpatient Services and Organization's Financial Outcomes, Multivariate Model, 2018–2020 RAND Prices eTable 3. Association Between Commercial-to-Medicare Price Ratio for Inpatient Services and Organization's Financial Outcomes, Multivariate Model, 2018–2020 RAND Prices (Unweighted) [file jamahealthforum-e225444-s001.pdf]

Supplemental Online Content

Blavin F, Kane N, Berenson R, Blanchfield B, Zuckerman S. Association of commercial-to-Medicare relative prices with health system financial performance. *JAMA Health Forum.* 2023;4(2):e225444. doi:10.1001/jamahealthforum.2022.5444

eAppendix. Comparison of Sample Characteristics to the Universe of Health Systems from the AHA

eTable 1. Characteristics of Analysis Sample vs AHA

eTable 2. Association Between Commercial-to-Medicare Price Ratio for Inpatient and Outpatient Services and Organization's Financial Outcomes, Multivariate Model, 2018–2020 RAND Prices

eTable 3. Association Between Commercial-to-Medicare Price Ratio for Inpatient Services and Organization's Financial Outcomes, Multivariate Model, 2018–2020 RAND Prices (Unweighted)

This supplemental material has been provided by the authors to give readers additional information about their work.

© 2023 Blavin F et al. *JAMA Health Forum.*

eAppendix. Comparison of Sample Characteristics to the Universe of Health Systems from the AHA

In the AHA Annual Survey Database, a multihospital health care system is an entity with two or more hospitals owned, leased, sponsored, or contract managed by a central organization. Multihospital systems, as defined in the analysis sample, include groups of two or more short-stay hospitals under joint ownership, linked by the Agency for Healthcare Research and Quality's (AHRQ) Compendium of U.S. Health Systems. AHRQ combines the AHA Annual Survey Database and IQVIA OneKey (a database used for sales/marketing purposes) to identify hospitals and link them to systems; these two data sources vary in ways that affect their characterization of hospitals and their linkages to systems.[i]

The first two columns of Appendix Table 1 compare the characteristics of multihospital health systems in our sample (141 out of 156 observations) with the universe of health systems in the AHA. The next two columns compare the characteristics of the 15 individual hospitals in our sample with the AHA universe of those not part of multihospital system. The final set of columns includes the combined averages, consistent with the health system characteristics in Table 2 of the main text.

Overall, our sample overrepresented larger, non-profit systems since they were more likely to have publicly available and easier to access audited financial statements relative to smaller for-profit systems and government-owned systems. Some key points from the table include the following:

- Our sample included nearly 40 percent (141 out of 357) of health systems in the AHA universe and only a few hospitals (15 or 1%) that are not part of a health system.
- The average system and individual hospital in our sample are larger than those in the full AHA universe e.g., 255,011 vs. 154,842 average adjusted admissions among systems and 12 vs. 8 hospitals per system on average.
- 92.2 percent of systems in our sample are predominantly non-profit compared with 78.8 percent of all systems in the AHA. However, as discussed in the main text, while for-profit health systems only accounted a small share of our sample, they represented a highly disproportionate share (approximately 2/3rd) of for-profit systems' adjusted admissions. Our sample also had a smaller share of government-owned systems relative the share in the full AHA universe.
- Relative to the AHA universe, our sample overrepresented systems in the northeast and underrepresented systems in the South.

© 2023 Blavin F et al. *JAMA Health Forum.*

eTable 1. Characteristics of Analysis Sample Versus AHA

	Multihospital systems		Not multi-hospital system		Combined average	
	Analysis Sample	AHA	Analysis Sample	AHA	Analysis Sample	AHA
**Number of observations**	141	357	15	1,471	156	1,828
**Total adjusted admissions**	36,000,000	55,300,000	269,800	15,900,000	36,269,800	71,200,000
**Average adjusted admissions**	255,011	154,842	17,987	10,797	232,499	38,950
**Total number of hospitals**	1,701	2,872	15	1,471	1,716	4,343
**Average number of hospitals**	12.1	8.0	1.0	1.0	11.0	2.4
**% adjusted admissions rural**	1.69%	3.12%	26.67%	39.90%	4.09%	32.72%
**Ownership**						
Non-profit	92.20%	78.71%	53.33%	47.45%	88.46%	53.56%
For-profit	2.84%	8.68%	6.67%	7.00%	3.21%	7.33%
Nonfederal govt	4.96%	12.61%	40.00%	45.55%	8.33%	39.11%
**Region**						
Midwest	26.95%	25.21%	13.33%	32.22%	25.64%	30.85%
Northeast	31.21%	19.33%	46.67%	11.28%	32.69%	12.86%
South	21.99%	38.94%	6.67%	35.15%	20.51%	35.89%
West	19.86%	16.53%	33.33%	21.35%	21.15%	20.40%

Notes: Total sample includes 156 observations, covering independent hospitals (*n*=15) and multihospital systems (*n*=141) that were included in round 4 of the RAND Hospital Price Transparency Study (hospital price data). This final sample excludes entities with missing payer mix or other financial metrics from the Audited Financial Statement data. AHA data are from the 2018 American Hospital Association Annual Survey Database.

© 2023 Blavin F et al. *JAMA Health Forum.*

eTable 2. Association Between Commercial-to-Medicare Price Ratio for Inpatient and Outpatient Services and Organization's Financial Outcomes, Multivariate Model, 2018–2020 RAND Prices

	(1)	(2)
	Days Cash on Hand[a]	Operating Margins
Relative price for inpatient and outpatient services	0.217***	2.59***
	(0.217 - 0.218)	(2.59 - 2.59)
Part of multihospital system	0.320***	-3.24***
	(0.317 - 0.323)	(-3.26 - -3.22)
Share of adjusted admissions that are rural	-0.017***	-5.41***
	(-0.021 - -0.014)	(-5.45 - -5.38)
For-profit ownership	N/A[a]	2.69***
		(2.69 - 2.69)
Government ownership	-0.147***	0.74***
	(-0.148 - -0.146)	(0.73 - 0.74)
Midwest	-0.056***	-0.81***
	(-0.056 - -0.055)	(-0.81 - -0.81)
Northeast	-0.675***	-0.72***
	(-0.676 - -0.674)	(-0.72 - -0.71)
South	-0.404***	-0.96***
	(-0.405 - -0.403)	(-0.96 - -0.96)
West	[Reference]	[Reference]
Medicaid %	-0.033***	-0.08***
	(-0.033 - -0.033)	(-0.08 - -0.08)
Constant	5.089***	1.21***
	(5.085 - 5.092)	(1.19 - 1.23)
Observations[b]	30,003,402	36,226,392
R-squared	0.32	0.31

95% confidence intervals are in parentheses

*** $p<0.01$

a: Estimates associated with for-profit ownership are missing because the DCOH sample excludes for-profit organizations.

b: Models are weighted by total adjusted admissions within the organization

eTable 3. Association Between Commercial-to-Medicare Price Ratio for Inpatient Services and Organization's Financial Outcomes, Multivariate Model, 2018–2020 RAND Prices (Unweighted)

	(1)	(2)
	Days Cash on Hand[a]	Operating Margins
Relative price for inpatient services	0.192*	1.33**
	(-0.003 - 0.388)	(0.30 - 2.35)
Part of multihospital system	0.482*	-1.56
	(-0.070 - 1.034)	(-4.39 - 1.28)
Share of adjusted admissions that are rural	0.697***	3.15
	(0.229 - 1.164)	(-2.44 - 8.75)
For-profit ownership	N/A[a]	0.62
		(-3.44 - 4.67)
Government ownership	0.016	-1.72
	(-0.355 - 0.388)	(-4.25 - 0.81)
Midwest	-0.112	-1.80**
	(-0.308 - 0.084)	(-3.38 - -0.21)
Northeast	-0.613***	-2.67***
	(-0.932 - -0.295)	(-4.40 - -0.94)
South	-0.371**	-1.88**
	(-0.660 - -0.082)	(-3.66 - -0.09)
West	[Reference]	[Reference]
Medicaid %	-0.023**	-0.05
	(-0.044 - -0.003)	(-0.12 - 0.02)
Adjusted Admissions	0.000	0.00
	(-0.000 - 0.000)	(-0.00 - 0.00)
Constant	4.851***	3.54**
	(4.156 - 5.546)	(0.06 - 7.02)
Observations	151	156
R-squared	0.29	0.16

95% confidence intervals are in parentheses

*** *p*<0.01, ** *p*<0.05, * *p*<0.1

a: Estimates associated with for-profit ownership are missing because the DCOH sample excludes for-profit organizations.

---

[i] Agency for Healthcare Research and Quality. Comparative Health System Performance Initiative: Compendium of U.S. Health Systems, 2018, Hospital Linkage File, Technical Documentation. AHRQ Publication No. 20-0010 November 2019 (updated January 2021). Agency for Healthcare Research and Quality, Rockville, MD.https://www.ahrq.gov/sites/default/files/wysiwyg/chsp/compendium/2018-hospital-linkage-techdoc-cx.pdf. Accessed December 5, 2022.

© 2023 Blavin F et al. *JAMA Health Forum.*
