# Supplementary material for: Association of Commercial-to-Medicare Relative Prices With Health System Financial Performance
Source: JAMA Health Forum. 2023 Feb 10;4(2):e225444. doi: 10.1001/jamahealthforum.2022.5444 (PMC9918880; doi:10.1001/jamahealthforum.2022.5444)
Supplement: Supplement 2. — Data Sharing Statement [file jamahealthforum-e225444-s002.pdf]

Data Sharing Statement

Blavin. Association of Commercial-to-Medicare Relative Prices With Health System Financial Performance. *JAMA Health Forum*. Published February 10, 2023.
doi:10.1001/jamahealthforum.2022.5444

Data
Data available: Yes
Data types: Data (not involving human participants), Data dictionary
How to access data: Data are available upon request. Please reach out to fblavin@urban.org for all inquiries.
When available: With publication

Supporting Documents
Document types: Statistical/analytic code
How to access documents: Please reach out to fblavin@urban.org for all inquiries.
When available: With publication

Additional Information
Who can access the data: Researchers requesting the data.
Types of analyses: For a specified purpose.
Mechanisms of data availability: With approval from project funder.
